# Electronic Medical Record Data Missingness and Interruption in Antiretroviral Therapy Among Adults and Children Living With HIV in Haiti: Retrospective Longitudinal Study

**DOI:** 10.2196/51574

**Published:** 2024-03-06

**Authors:** Andrew M Secor, Kemar Célestin, Margareth Jasmin, Jean Guy Honoré, Anjuli D Wagner, Kristin Beima-Sofie, Jillian Pintye, Nancy Puttkammer

**Affiliations:** 1 Department of Global Health, University of Washington, Seattle, WA, United States; 2 Centre Haïtien pour le Renforcement du Système de Santé, Port-au-Prince, Haiti; 3 International Training and Education Center for Health, Seattle, WA, United States

**Keywords:** HIV, Haiti, pediatrics, combination antiretroviral therapy, electronic medical record, data quality, child, children, antiretroviral, therapy, longitudinal study, HIV diagnosis, diagnosis, HIV care, patient records, quality of care, treatment, engagement

## Abstract

**Background:**

Children (aged 0-14 years) living with HIV often experience lower rates of HIV diagnosis, treatment, and viral load suppression. In Haiti, only 63% of children living with HIV know their HIV status (compared to 85% overall), 63% are on treatment (compared to 85% overall), and 48% are virally suppressed (compared to 73% overall). Electronic medical records (EMRs) can improve HIV care and patient outcomes, but these benefits are largely dependent on providers having access to quality and nonmissing data.

**Objective:**

We sought to understand the associations between EMR data missingness and interruption in antiretroviral therapy treatment by age group (pediatric vs adult).

**Methods:**

We assessed associations between patient intake record data missingness and interruption in treatment (IIT) status at 6 and 12 months post antiretroviral therapy initiation using patient-level data drawn from iSanté, the most widely used EMR in Haiti. Missingness was assessed for tuberculosis diagnosis, World Health Organization HIV stage, and weight using a composite score indicator (ie, the number of indicators of interest missing). Risk ratios were estimated using marginal parameters from multilevel modified Poisson models with robust error variances and random intercepts for the facility to account for clustering.

**Results:**

Data were drawn from 50 facilities and comprised 31,457 patient records from people living with HIV, of which 1306 (4.2%) were pediatric cases. Pediatric patients were more likely than adult patients to experience IIT (n=431, 33% vs n=7477, 23.4% at 6 months; *P*<.001). Additionally, pediatric patient records had higher data missingness, with 581 (44.5%) pediatric records missing at least 1 indicator of interest, compared to 7812 (25.9%) adult records (*P*<.001). Among pediatric patients, each additional indicator missing was associated with a 1.34 times greater likelihood of experiencing IIT at 6 months (95% CI 1.08-1.66; *P*=.008) and 1.24 times greater likelihood of experiencing IIT at 12 months (95% CI 1.05-1.46; *P*=.01). These relationships were not statistically significant for adult patients. Compared to pediatric patients with 0 missing indicators, pediatric patients with 1, 2, or 3 missing indicators were 1.59 (95% CI 1.26-2.01; *P*<.001), 1.74 (95% CI 1.02-2.97; *P*=.04), and 2.25 (95% CI 1.43-3.56; *P*=.001) times more likely to experience IIT at 6 months, respectively. Among adult patients, compared to patients with 0 indicators missing, having all 3 indicators missing was associated with being 1.32 times more likely to experience IIT at 6 months (95% CI 1.03-1.70; *P*=.03), while there was no association with IIT status for other levels of missingness.

**Conclusions:**

These findings suggest that both EMR data quality and quality of care are lower for children living with HIV in Haiti. This underscores the need for further research into the mechanisms by which EMR data quality impacts the quality of care and patient outcomes among this population. Efforts to improve both EMR data quality and quality of care should consider prioritizing pediatric patients.

## Introduction

Despite improvements in HIV testing, care, and treatment and reduced HIV incidence over the last 3 decades, Haiti has the largest population of people living with HIV in the Caribbean, with an estimated 1.8% of the population (150,000 persons) having received a positive HIV diagnosis, including nearly 6000 children (aged 0-14 years) living with HIV [1]. Children living with HIV often have lower rates of HIV diagnosis, treatment, and viral load suppression [2]. In Haiti, children living with HIV fare worse across all steps of the care cascade, with only 63% knowing their HIV status (compared to 85% overall), 63% on treatment (compared to 85% overall), and 48% virally suppressed (compared to 73% overall) [1]. Reviews of patient records in Haiti revealed that children living with HIV were significantly less likely to initiate antiretroviral therapy (ART) in a timely manner as compared to adults, and once initiated, were less likely than adults to be retained in ART treatment [3,4].

Electronic medical records (EMRs) can improve HIV patient care and outcomes in multiple ways, including (1) directly informing individual patient care, such as tracking clinical outcomes, ART adherence and retention, as well as patient follow-up; and (2) promoting provider compliance with treatment and care guidelines [5-13]. However, these benefits are largely dependent on providers having access to high-quality data (ie, reliable, timely, and nonmissing data) [14-16]. In the context of EMRs, data missingness is both an element of quality of care (vis-à-vis noncompliance with reporting guidelines) and can itself lead to lower quality of care, as missing data cannot be used to inform clinical decision-making [17]. However, despite the importance of data quality in the value proposition of EMRs, the evidence base exploring the association between data missingness and patient outcomes is limited, especially in resource-limited settings. Although many studies of EMRs include both data quality and patient outcomes as indicators of interest, a direct association between the two is rarely assessed. In addition, no studies reviewed for this paper assessed this relationship by age cohort.

We hypothesize that data missingness will be associated with greater interruption in treatment (IIT) and that this relationship may be larger among children living with HIV. We used ART patient data extracted from the iSanté EMR system to assess the association between age group, data missingness, and IIT.

## Methods

### Study Design

This was a retrospective longitudinal study using patient-level routine EMR data.

### Data Source

We used patient-level clinical and pharmacy data extracted from iSanté—the most widely used EMR in Haiti, which covers over 1.8 million primary care patients and more than 200,000 unique records for people living with HIV [18,19]. iSanté records include data on key HIV care cascade processes (eg, clinical history) and electronic pharmacy data (eg, ART dispensing and continuation).

### Sample

This analysis included data from 50 facilities and covered individuals who initiated ART between June 2016 and December 2021. Our analyses had a number of data exclusions. A total of 24 facilities were excluded from the analysis due to being prison-based facilities, having >20% of records entered more than 90 days after the visit date, or having a mean number of prescription records per patient less than 5 (suggesting data record input issues). Data before June 2016 were excluded to account for changes in treatment patterns following adoption of the test-and-treat approach to HIV care in mid-2016. Patient records included in the analysis were restricted to patients who initiated ART at least *n+2* months before the data extraction date (end of July 2022) to allow for sufficient follow-up time to observe the outcome and to account for any delay in the entry of patient files, where *n* refers to the 6- or 12-month IIT outcome (eg, for the 6 months outcome, data were restricted to those who had initiated ART before December 2021). Individuals without date of birth data (n=1174, 3.2%) were excluded as correct age group categorization was essential for the analysis. Additionally, to better assess the relationship between data missingness at intake and IIT status 6 or 12 months after initiating ART, patients who completed their intake visit more than 3 months prior to initiating ART were excluded from the analysis (n=4083, 11.5%). A CONSORT (Consolidated Standards of Reporting Trials) flow diagram can be found in Multimedia Appendix 1 [20].

### Conceptual Model

**Figure 1.**
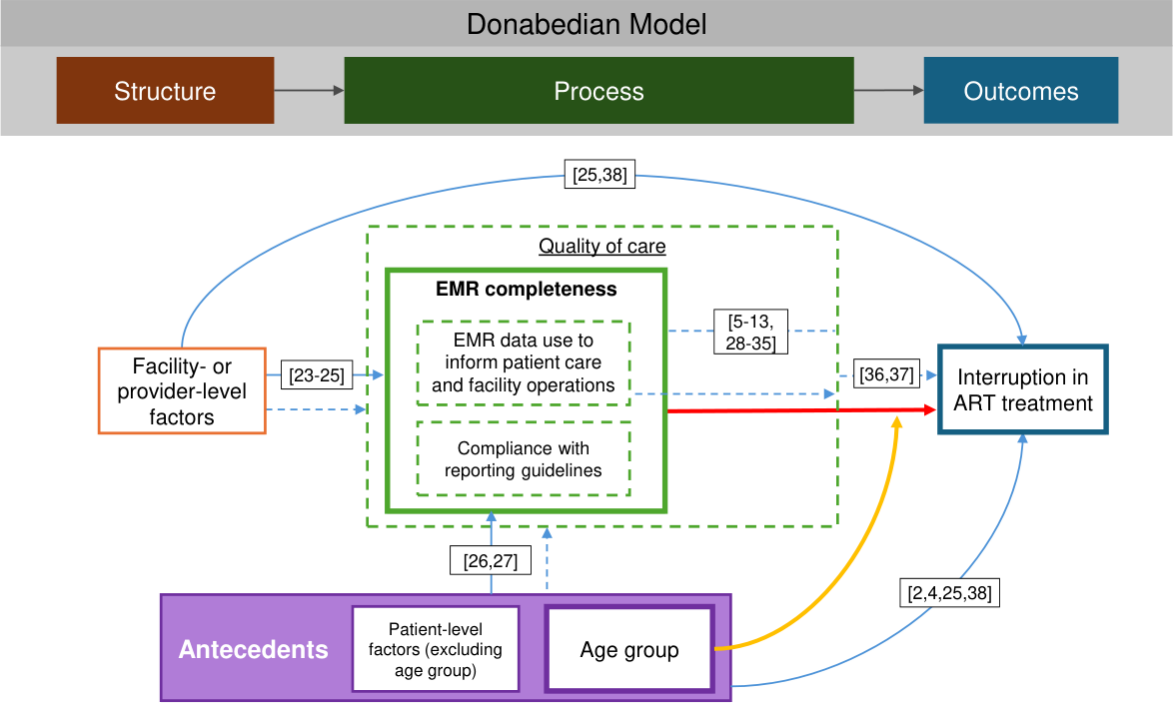
Conceptual model [2,4-13,23-38]. The blue lines indicate proposed causal pathways; the red line indicates the observed association of interest; the yellow line indicates effect modification by age; the dashed lines indicate unobserved pathways or variables. ART: antiretroviral therapy; EMR: electronic medical record.

Figure 1 shows our proposed causal model, which is situated within the Donabedian framework [21] for quality of care, as modified by Coyle and Battles [22] to include medical antecedents. The Donabedian framework divides care into 3 primary components: structure (ie, the context in which care is delivered), process (ie, actual service delivery), and outcome. In the context of HIV care and EMRs, these can be understood as the facility or organizational context in which HIV care is delivered as well as the system aspects of EMRs (eg, accessibility and usability); the provision of HIV care, including the use of EMRs to both document and inform care; and HIV outcomes (eg, IIT). The various pathways in our conceptual model were justified through the published literature [2,4-13,23-38].

### Analysis

#### Outcome Variable

The primary outcomes of interest were IIT at the current facility at 6 and 12 months post ART initiation, defined as being more than 28 days late in picking up ART medication as of the dates 6 or 12 months after initiating ART. This definition for IIT status has been used in prior research in Haiti and other settings [39-41].

#### Covariates

Age groups were categorized as pediatric (0-14 years) and adult (>15 years) as of the time of ART initiation, following the age definition used to define pediatric care in Haiti (<15 years).

#### Data Missingness

Data missingness was defined as an indicator not being collected during the patient’s intake visit. Assessment of missingness was restricted to indicators that were shared between both pediatric and adult intake forms, indicators that were clinically meaningful for HIV care, and where missingness could be differentiated from the absence of that issue (eg, the headache symptomology field may be missing due to a patient not presenting with a headache or due to the provider failing to document that issue, whereas the World Health Organization [WHO] HIV stage indicator is expected to be completed for all patients) [42]. Within these stipulations, we assessed missingness for weight, current WHO HIV stage, and current tuberculosis (TB) diagnosis. As the importance and impact of missingness for particular indicators may vary by age group (eg, routine documentation of weight is generally of higher importance for pediatric patients), missingness was analyzed as individual binary outcomes (defined as missing or nonmissing) as well as through a composite score indicator (the number of indicators of interest missing), which was analyzed as both a continuous and categorical outcome.

### Models

Associations between data missingness (exposure) and interruption in ART treatment at a patient’s current facility (outcome) were assessed through marginal parameters from multilevel modified Poisson models with robust error variances and random intercepts for the facility to account for clustering. Modified Poisson models have been shown to provide unbiased estimates of the risk ratio, important for nonrare binary outcomes where odds ratios estimated through logistic regression will overestimate the risk ratio and potentially lead to improper interpretation of the results [43,44]. Patient sex, facility type, ownership, patient volume, and duration of iSanté use were included as fixed effects to control for potential confounding. Models were stratified by age group to understand the relationship between data missingness and IIT status within each age group. Additional models were run with the age group as an interaction term with the continuous composite indicator to assess the statistical significance of the age group as an effect modifier in the association between missingness and IIT status.

### Ethical Considerations

The secondary use of deidentified patient data from the iSanté EMR was approved by the University of Washington Human Subjects Division as nonengaged research (STUDY00016591 “Patient Risk Profiles for Interruption in Treatment among People Living with HIV in Haiti: Leveraging Health Information Systems and Prediction Models to Identify Patients at High Risk”). The research was also reviewed and approved by the Haiti Ministry of Public Health and Population’s National Bioethics Committee (reference number 2223-26).

## Results

### Primary Findings

In total, data were drawn from 50 facilities across 9 departments (of 10 total) in Haiti and comprised 31,457 patient records for people living with HIV. Of these, 30,151 (95.8%) were adult patients and 1306 (4.2%) were pediatric patients. The majority of patients (n=19,544, 62.1%) were female and received care at health centers (n=19,051, 60.6%) or hospitals (n=9883, 31.4%). The median duration of iSanté use at each health facility was 17.5 (IQR 15.8-18.3) years, and the median monthly patient volume was 348 (IQR 172-544). Table 1 further details participant and facility characteristics.

**Table 1.** Participant characteristics^a^.

Characteristics		Patients					
		Overall (N=31,457)		Adult (n=30,151, 95.8%)		Pediatric (n=1306, 4.2%)	
**Sex, n (%)**							
	Female	19,544 (62.1)		18,855 (62.5)		689 (52.8)	
	Male	11,913 (37.9)		11,296 (37.5)		617 (47.2)	
Age (years), median (IQR)		35 (27-44)		35 (28-44)		3 (0-9)	
**Facility type, n (%)**							
	Health center	19,051 (60.6)		18,319 (60.8)		732 (56.0)	
	Hospital	9883 (31.4)		9406 (31.2)		477 (36.5)	
	Dispensary	2523 (8.0)		2426 (8.0)		97 (7.4)	
**Facility ownership, n (%)**							
	Both public and private	7337 (23.3)		7008 (23.2)		329 (25.2)	
	Private	11,466 (36.4)		11,109 (36.8)		357 (27.3)	
	Public	12,654 (40.2)		12,034 (39.9)		620 (47.5)	
Duration of iSanté use, median (IQR)		17.5 (15.8-18.3)		17.5 (15.3-18.3)		18.1 (17.0-18.3)	
Monthly patient volume, median (IQR)		348 (172-544)		348 (172-544)		408 (200-626)	

a Facility-related characteristics are described at the patient level (eg, the proportion of patients initiating antiretroviral therapy at a health center versus a hospital or dispensary).

IIT status and indicator missingness are detailed in Table 2. Across all age groups, the proportion of patients who experienced IIT at 6 and 12 months post ART initiation were 23.8% (n=7477) and 29.3% (n=9222), respectively. Overall, the weight indicator had the highest level of missingness, with 5365 (17.1%) patient records missing weight data, while TB diagnosis had the lowest (n=1417, 4.5%). Both IIT status and data missingness were higher among pediatric patients. Pediatric patients were more likely than adult patients to be IIT at both 6 months (n=431, 33.0% vs n=7046, 23.4%; *P*<.001) and 12 months (n=551, 42.2% vs n=8671, 28.8%; *P*<.001). Only 55.5% (n=725) of pediatric patient records had no indicators of interest missing, compared to 74.1% (n=22,339) of adult patient records. Pediatric patient records were also more likely to have at least 3 (3.1%) indicators missing compared to adult records (n=401, 1.3%; *P*<.001). Variation in missingness across age groups was greatest for the WHO HIV stage, with 32.5% (n=425) of pediatric records missing this indicator compared to 11.1% (n=3355) of adult records (*P*<.001).

**Table 2.** Interruption in treatment (IIT) status and indicator missingness.

Characteristics		Patients				*P* value^a^
		Overall (N=31,457), n (%)		Pediatric (n=1306), n (%)		
**IIT status**						
	6 months	7477 (23.8)		431 (33.0)		<.001
	12 months	9222 (29.3)		551 (42.2)		<.001
**Indicator missingness**						
	Weight	5365 (17.1)		265 (20.3)		.001
	WHO^b^ HIV stage	3780 (12.0)		425 (32.5)		<.001
	TB^c^ diagnosis	1417 (4.5)		79 (6.0)		.006
**Composite missingness score (number of indicators missing)**						
	0	23,064 (73.3)		725 (55.5)		<.001
	1	6666 (21.2)		434 (33.2)		<.001
	2	1285 (4.1)		106 (8.1)		<.001
	3	442 (1.4)		41 (3.1)		<.001

a Pearson *χ*2 test.

b WHO: World Health Organization.

c TB: tuberculosis.

**Figure 2.**
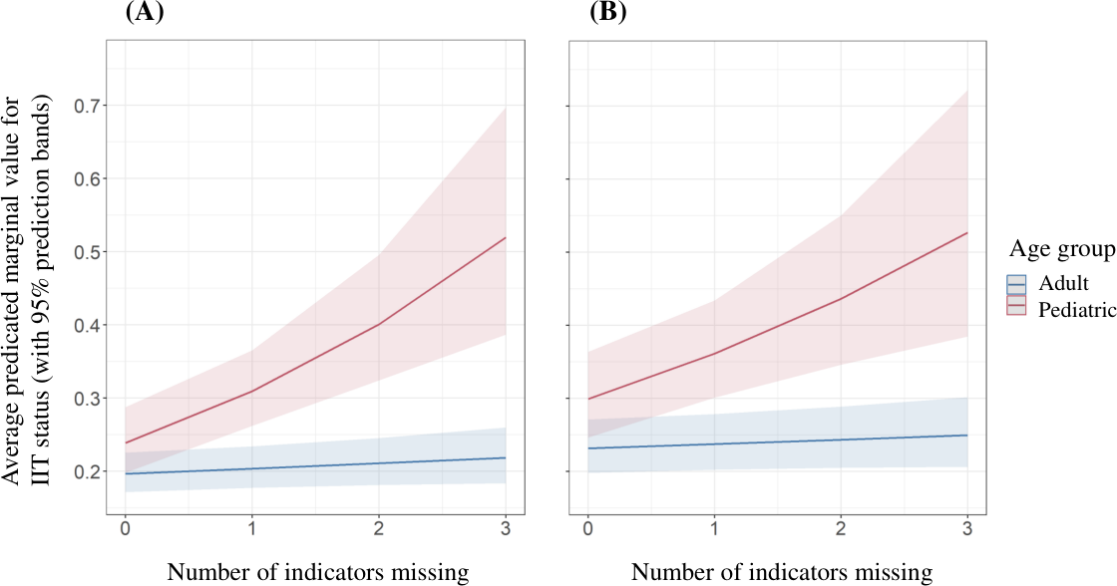
Multivariable regression of interruption in treatment (IIT) status at (A) 6 months post antiretroviral therapy (ART) initiation and (B) 12 months post ART initiation against composite missingness score (continuous), stratified by age group.

Results from models exploring the association between IIT status and the composite missingness score as a continuous variable are shown in Figure 2 (full multivariable regression results can be found in Multimedia Appendix 2). Statistically significant associations were observed between higher values of the composite missingness score and a greater likelihood of experiencing IIT at both 6 and 12 months among pediatric patients. However, no such association was observed among adult patients for either outcome. Among pediatric patients, each additional indicator missing was associated with a 1.34 times greater likelihood of experiencing IIT at 6 months post ART initiation (95% CI 1.08-1.66; *P*=.008) and 1.24 times greater likelihood of experiencing IIT at 12 months (95% CI 1.05-1.46; *P*=.01). Our interaction models (not shown) revealed that the relationship between the composite score indicator and IIT status was statistically significantly larger among pediatric patients compared to adult patients at both 6 months, where pediatric patients had a 25% greater risk of experiencing IIT for each additional missing element compared to adult patients (95% CI 1.02-1.53; *P*=.03), and 12 months, where pediatric patients had an 18% greater risk of experiencing IIT for each additional missing element compared to adult patients (95% CI 1.01-1.38; *P*=.04).

**Figure 3.**
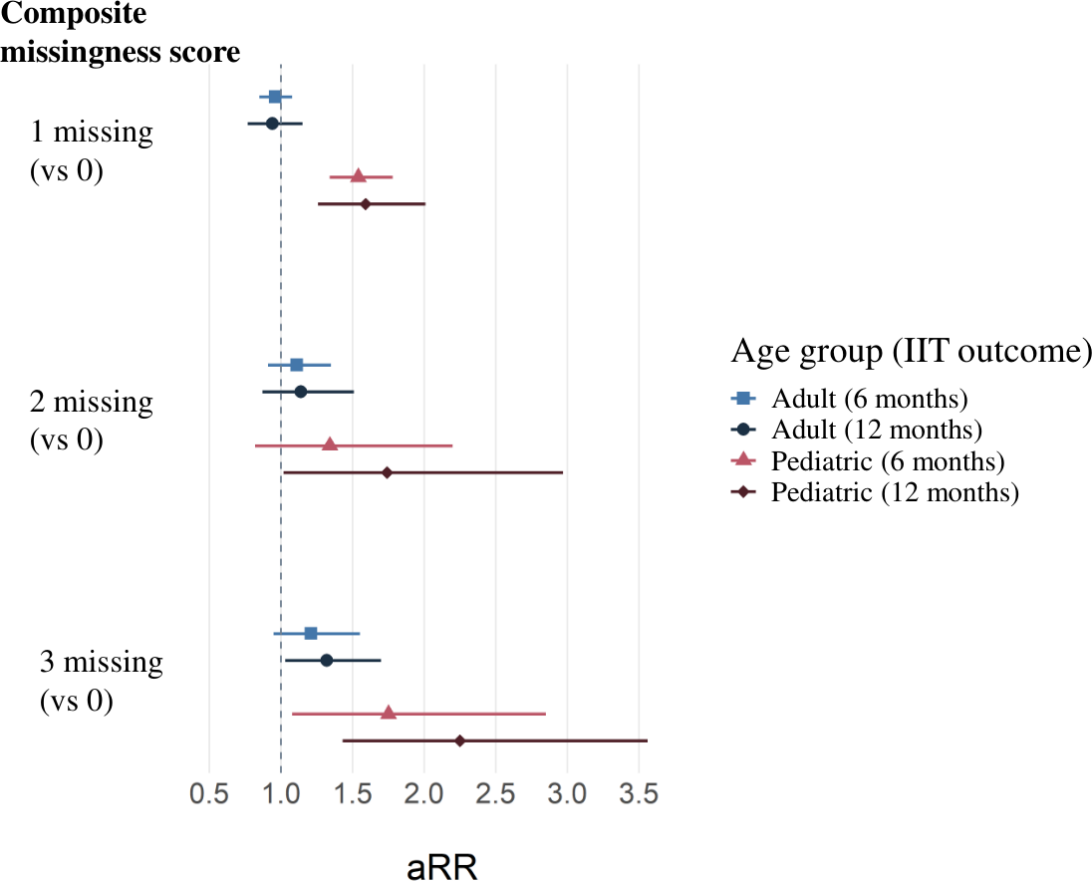
Multivariable regression of interruption in treatment (IIT) status at 6 and 12 months post antiretroviral therapy initiation against composite missingness score (categorical), stratified by age group. aRR: adjusted risk ratio.

We also assessed the composite missingness score as a categorical variable (Figure 3) to understand the estimates of excess risk in the absence of the assumption of a linear relationship between missingness and IIT status (full multivariable regression results can be found in Multimedia Appendix 2). Compared to pediatric patients with 0 missing indicators, pediatric patients with 1, 2, or 3 missing indicators were 1.59 (95% CI 1.26-2.01; *P*<.001), 1.74 (95% CI 1.02-2.97; *P*=.04), and 2.25 (95% CI 1.43-3.56*; P*=.001) times more likely to experience IIT at 6 months, respectively. At 12 months, pediatric patients with 1, 2, or 3 missing indicators were 1.54 (95% CI 1.34-1.78; *P*<.001), 1.34 (95% CI 0.82-2.20; *P*=.24), and 1.75 (95% CI 1.08-2.85; *P*=.02) times more likely to experience IIT, respectively, although this association was no longer significant for those with 2 indicators missing. Among adult patients, compared to patients with 0 indicators missing, having all 3 indicators missing was associated with being 1.32 times more likely to experience IIT at 6 months (95% CI 1.03-1.70; *P*=.03), while having 3 indicators missing was not associated with IIT at 12 months, and having 1 or 2 indicators missing was not associated with IIT at either 6 or 12 months.

For the individual missingness indicators (Multimedia Appendix 2), only the WHO HIV stage indicator was associated with IIT status among pediatric patients, where pediatric patients with missing WHO HIV stage data on their intake form were 2.17 times more likely to experience IIT at 6 months (95% CI 1.79-2.64; *P*<.001) and 1.79 times more likely to experience IIT at 12 months (95% CI 1.54-2.08; *P*<.001), as compared to pediatric patients with nonmissing WHO HIV stage data. Missingness for the WHO stage data among adult patients and missingness for weight and TB status among either age group were not associated with IIT status at either 6 or 12 months.

### Sensitivity Analyses

We hypothesized that providers at facilities with a lower proportion of pediatric patients may be less familiar with pediatric care, and therefore, less compliant with treatment and reporting guidelines, which could potentially impact the relationship between data missingness and IIT. However, neither missingness nor IIT status showed a significant association with the proportion of pediatric patients at a given facility, and an interaction model did not show any difference in the relationship between data missingness and IIT status by the proportion of pediatric patients (data are not shown). We additionally explored a more granular definition for age groups 0-9, 10-14, 15-19, 20-24, and >25 years. In age group–stratified models, the association between the continuous composite data missingness score and IIT status at 6 months was only statistically significant for the 0-9 age group, which showed a positive association between greater missingness and likelihood of experiencing IIT (data are not shown).

## Discussion

### Principal Findings

In this retrospective longitudinal study of patient record data drawn from the iSanté EMR system in Haiti, we found that both data missingness and interruption in ART treatment were higher for pediatric patients compared to adult patients; nearly one-third of pediatric patients had IIT at 6 months compared to just over one-fifth of adults, and nearly half of pediatric patients had missing values for indicators of interest on their intake forms compared to just over one-quarter of adult patients. Data missingness showed a substantial and significant association with greater IIT, with adult patients being 30% more likely and pediatric patients more than twice as likely to have IIT at 6 months when all 3 indicators of interest were missing. The relationship between missingness and IIT status was stronger and more consistent among pediatric patients; pediatric patients showed statistically significantly greater likelihood of experiencing IIT at 6 and 12 months for the composite score indicator both overall (continuous) and across all levels of missingness (categorical), while for adult patients this relationship was only significant at 6 months and for the highest level of missingness in the categorical analysis. Individual indicator missingness showed little association with IIT status, except for the WHO HIV stage among pediatric patients. Within the modified Donabedian quality of care framework, our results show a link between the process of care provision (vis-à-vis compliance with reporting guidelines and data use for clinical decision-making) and patient outcomes (IIT status) after adjusting for structural elements (ie, facility characteristics), with the association being highly dependent on medical care antecedents (ie, patient age group) [21,22].

There is a rich evidence base showing the potential impact of EMR use on HIV service provision and quality of care by promoting adherence to care guidelines, enabling higher quality patient data, improving provider efficiency, and informing patient care, tracking, and follow-up [5-13]. The benefits of EMRs, however, are largely predicated on providers having access to quality data (ie, reliable, timely, and nonmissing) to inform their work, and there is a growing evidence base on the importance and impact of patient record quality (electronic or otherwise) on quality of care, care engagement, or health outcomes [14-16,29,30]. Particularly relevant to this analysis, one study of more than 6000 patient records collected from the National Clinical Audit for Rheumatoid and Early Inflammatory Arthritis found that missing baseline patient data was significantly associated with the odds of timely initiation of treatment being halved [29]. In a qualitative study of health care professionals in South Africa, participants reported viewing data quality as an critical element in the provision of quality health care services, including how poor EMR information integrity can lead “to errors that endanger patient safety or decrease the quality of care” [45]. A systematic review found that data missingness was a commonly cited barrier to the use of EMRs to inform population health efforts [12]. In another systematic review, Albagmi [11] found that EMRs were associated with both better documentation and higher quality of care, although a direct causal relationship between data quality and quality of care was not directly assessed. This limitation is common to much of the literature on EMR data quality; many studies of EMR implementation include both data quality and quality of care indicators as outcomes or indicators of interest, but few directly assess the relationship between data quality and quality of care or patient outcomes. Our results, therefore, contribute to this limited evidence base, providing evidence that EMR data quality is associated with interruption in ART treatment.

Although we have established a temporal sequence for the relationship between data quality and IIT status, the absence of measurements for other elements of quality of care makes it impossible to discern whether the observed association was due to poor data quality itself or data quality as a proxy for broader quality of care. Data quality could be a marker of lower provider competence, poorer supplies and infrastructure at the health facility, higher provider-patient ratio, lower contact time between providers and patients, or other phenomena associated with IIT status. Further research is necessary to understand the role data missingness plays in care provision.

Our finding that overall missingness was higher among pediatric patients and that the association between missingness and IIT status was stronger among pediatric patients supports our hypothesis that there may be differential quality of care among pediatric patients leading to poorer retention in care. Pediatric populations living with HIV have unique care needs, and poorer engagement for pediatric patients across the HIV care cascade is a multifaceted issue, involving behavioral, psychosocial, pharmacokinetic, and structural factors [17,46]. The literature has identified a number of key barriers to pediatric ART adherence, including stigma among caregivers to seek or continue care for children; lack of education or training for caregivers on caring for a child living with HIV; complexities inherent to a patient-caregiver-provider relationship; limited patient agency due to age and patient-caregiver power structures; patient-led treatment refusal, sometimes due to a lack of palatable formulations for younger patients; and lack of providers trained in pediatric HIV care or family-based service delivery [17,47,48]. Relevant to this analysis, prior research has shown direct links between quality of care, care engagement, and patient outcomes for this population. In their analysis of children living with HIV in Nigeria, Ojikutu et al [37] found that higher quality care—measured as a composite score exploring TB screening, adherence measurement and counseling, CD4 and weight documentation, and medication prescription—was significantly associated with a lower likelihood of pediatric and adolescent patients being lost to follow-up and mortality. Improving the quality of care for children living with HIV, including better patient record quality, is necessary to address the gaps in HIV testing and treatment among children living with HIV.

### Limitations

At present, it is not possible to track patients between facilities within our analysis data. As such, it is not possible to distinguish patients who transferred to a new facility but remained on ART and those who interrupted or fully discontinued treatment. As a result, our ART retention outcome was defined as an IIT at a patient’s current facility rather than interruption overall. This outcome still fits within our causal model, with lower quality of care being feasibly associated with either an actual IIT or transfer to another facility for higher quality care, and it still represents a meaningful proxy indicator for clinical outcomes, as facility transfer may be associated with ART treatment gaps or discontinuation. Patient transfers are also not a limitation specific to this study; a systematic review of ART retention studies found that nearly 20% of patients classified as lost to follow-up had actually self-transferred to another facility [49].

Additionally, our results may be confounded if the missingness of the indicators is associated with the values of that indicator as well as our outcome. For example, if a higher WHO HIV stage is associated with both a greater likelihood of being missing and a greater likelihood of IIT, the observed association may be due to the latent WHO HIV stage rather than the data missingness. Of note, although integrated with iSanté, pharmacy data used to calculate the IIT outcome variables are collected through different mechanisms and staff. This includes greater data quality oversight, in part due to their inclusion in routine President’s Emergency Plan for AIDS Relief (PEPFAR) monitoring, evaluation, and reporting. Therefore, we do not anticipate that misclassification of the IIT outcome due to missing pharmacy data will be highly correlated with our exposure (missingness among indicators of interest), and thus, it will not present a substantial risk of bias. Finally, we were not able to assess associations with clinical outcomes (eg, viral suppression) due to data availability limitations.

### Strengths

This was an observational study, and therefore, it could not assess a causal relationship between data missingness and IIT; however, our hypothesis is strengthened by the robust sample size and analytical design; strong association observed between missingness and IIT status; a dose-response relationship wherein greater missingness was associated with greater likelihood of a patient having IIT; and established temporal sequence, as the intake data are completed prior to ART initiation and the IIT outcomes.

### Conclusions

Our analysis showed that both patient record data missingness for key indicators and interruption in ART treatment were common among patients, with nearly one-quarter of patients having IIT at 6 months and more than one-quarter of patients missing at least 1 indicator of interest in their patient record. Both IIT status and data missingness were more common among pediatric patients. Greater data missingness was associated with a higher likelihood of being IIT at 6 and 12 months for both pediatric and adult patients, although the association was stronger and more consistent among pediatric patients. Our findings motivate further research into the mechanisms by which EMR data quality impacts the quality of care and patient outcomes, particularly among children living with HIV. Additionally, efforts to improve both EMR data quality and quality of care should consider prioritizing pediatric patients.

## Supplementary material

10.2196/51574 Multimedia Appendix 1 The CONSORT (Consolidated Standards of Reporting Trials) flow diagram.

10.2196/51574 Multimedia Appendix 2 Multivariable regression tables.

## Abbreviations

ART: antiretroviral therapy
CONSORT: Consolidated Standards of Reporting Trials
EMR: electronic medical record
IIT: interruption in treatment
PEPFAR: President’s Emergency Plan for AIDS Relief
TB: tuberculosis
WHO: World Health Organization

## References

[1] Joint United Nations Programme on HIV/AIDS (UNAIDS) (2022). UNAIDS data 2022. UNAIDS.

[2] Abelman R, Alons C, Stockman J (2020). Implementation of differentiated service delivery for paediatric HIV care and treatment: opportunities, challenges and experience from seven sub-Saharan African countries. Fam Med Community Health.

[3] Puttkammer N, Parrish C, Desir Y (2021). Timely initiation of HIV antiretroviral therapy in Haiti 2004-2018: a retrospective cohort study. Rev Panam Salud Publica.

[4] Auld AF, Pelletier V, Robin EG (2017). Retention throughout the HIV care and treatment cascade: from diagnosis to antiretroviral treatment of adults and children living with HIV-Haiti, 1985-2015. Am J Trop Med Hyg.

[5] Gatiti P, Ndirangu E, Mwangi J, Mwanzu A, Ramadhani T (2021). Enhancing healthcare quality in hospitals through electronic health records: a systematic review. J Health Inform Dev Ctries.

[6] Campanella P, Lovato E, Marone C (2016). The impact of electronic health records on healthcare quality: a systematic review and meta-analysis. Eur J Public Health.

[7] Holroyd-Leduc JM, Lorenzetti D, Straus SE, Sykes L, Quan H (2011). The impact of the electronic medical record on structure, process, and outcomes within primary care: a systematic review of the evidence. J Am Med Inform Assoc.

[8] Jawhari B, Ludwick D, Keenan L, Zakus D, Hayward R (2016). Benefits and challenges of EMR implementations in low resource settings: a state-of-the-art review. BMC Med Inform Decis Mak.

[9] Oluoch T, Santas X, Kwaro D (2012). The effect of electronic medical record-based clinical decision support on HIV care in resource-constrained settings: a systematic review. Int J Med Inform.

[10] Oluoch T, Kwaro D, Ssempijja V (2015). Better adherence to pre-antiretroviral therapy guidelines after implementing an electronic medical record system in rural Kenyan HIV clinics: a multicenter pre-post study. Int J Infect Dis.

[11] Albagmi S (2021). The effectiveness of EMR implementation regarding reducing documentation errors and waiting time for patients in outpatient clinics: a systematic review. F1000Res.

[12] Kruse CS, Stein A, Thomas H, Kaur H (2018). The use of electronic health records to support population health: a systematic review of the literature. J Med Syst.

[13] Chaudhry B, Wang J, Wu S (2006). Systematic review: impact of health information technology on quality, efficiency, and costs of medical care. Ann Intern Med.

[14] Ndabarora E, Chipps JA, Uys L (2014). Systematic review of health data quality management and best practices at community and district levels in LMIC. Inf Dev.

[15] Pirkle CM, Dumont A, Zunzunegui M-V (2012). Medical recordkeeping, essential but overlooked aspect of quality of care in resource-limited settings. Int J Qual Health Care.

[16] Arts DGT, De Keizer NF, Scheffer GJ (2002). Defining and improving data quality in medical registries: a literature review, case study, and generic framework. J Am Med Inform Assoc.

[17] Panel on Antiretroviral Therapy and Medical Management of Children Living with HIV (2023). Guidelines for the use of antiretroviral agents in pediatric HIV infection. Clinical Info HIV.

[18] deRiel E, Puttkammer N, Hyppolite N (2018). Success factors for implementing and sustaining a mature electronic medical record in a low-resource setting: a case study of iSanté in Haiti. Health Policy Plan.

[19] Bardfield J, Agins BD, Jasmin M, Celestin N, Duval N, Balan JG, Marquez LR (2020). Improving Health Care in Low- and Middle-Income Countries: A Case Book.

[20] Schulz KF, Altman DG, Moher D, CONSORT Group (2010). CONSORT 2010 statement: updated guidelines for reporting parallel group randomised trials. BMJ.

[21] Donabedian A (2005). Evaluating the quality of medical care. 1966. Milbank Q.

[22] Coyle YM, Battles JB (1999). Using antecedents of medical care to develop valid quality of care measures. Int J Qual Health Care.

[23] Puttkammer N, Baseman JG, Devine EB (2016). An assessment of data quality in a multi-site electronic medical record system in Haiti. Int J Med Inform.

[24] van der Bij S, Khan N, Veen PT, de Bakker DH, Verheij RA (2017). Improving the quality of EHR recording in primary care: a data quality feedback tool. J Am Med Inform Assoc.

[25] Forster M, Bailey C, Brinkhof MWG (2008). Electronic medical record systems, data quality and loss to follow-up: survey of antiretroviral therapy programmes in resource-limited settings. Bull World Health Organ.

[26] Cook LA, Sachs J, Weiskopf NG (2021). The quality of social determinants data in the electronic health record: a systematic review. J Am Med Inform Assoc.

[27] Terry AL, Stewart M, Cejic S (2019). A basic model for assessing primary health care electronic medical record data quality. BMC Med Inform Decis Mak.

[28] DesRoches CM, Campbell EG, Rao SR (2008). Electronic health records in ambulatory care--a national survey of physicians. N Engl J Med.

[29] Yates M, Bechman K, Dennison EM (2020). Data quality predicts care quality: findings from a national clinical audit. Arthritis Res Ther.

[30] Grissinger MC, Hicks RW, Keroack MA, Marella WM, Vaida AJ (2010). Harmful medication errors involving unfractionated and low-molecular-weight heparin in three patient safety reporting programs. Jt Comm J Qual Patient Saf.

[31] Oluoch T, Katana A, Ssempijja V (2014). Electronic medical record systems are associated with appropriate placement of HIV patients on antiretroviral therapy in rural health facilities in Kenya: a retrospective pre-post study. J Am Med Inform Assoc.

[32] Oluoch T, Katana A, Kwaro D (2016). Effect of a clinical decision support system on early action on immunological treatment failure in patients with HIV in Kenya: a cluster randomised controlled trial. Lancet HIV.

[33] Oluoch T, Cornet R, Muthusi J (2021). A clinical decision support system is associated with reduced loss to follow-up among patients receiving HIV treatment in Kenya: a cluster randomized trial. BMC Med Inform Decis Mak.

[34] Matheson AI, Baseman JG, Wagner SH (2012). Implementation and expansion of an electronic medical record for HIV care and treatment in Haiti: an assessment of system use and the impact of large-scale disruptions. Int J Med Inform.

[35] King J, Patel V, Jamoom EW, Furukawa MF (2014). Clinical benefits of electronic health record use: national findings. Health Serv Res.

[36] Hargreaves S, Rustage K, Nellums LB (2019). Do quality improvement initiatives improve outcomes for patients in antiretroviral programs in low- and middle-income countries? A systematic review. J Acquir Immune Defic Syndr.

[37] Ojikutu B, Higgins-Biddle M, Greeson D (2014). The association between quality of HIV care, loss to follow-up and mortality in pediatric and adolescent patients receiving antiretroviral therapy in Nigeria. PLoS One.

[38] Frijters EM, Hermans LE, Wensing AMJ, Devillé W, Tempelman HA, De Wit JBF (2020). Risk factors for loss to follow-up from antiretroviral therapy programmes in low-income and middle-income countries. AIDS.

[39] Puttkammer N, Parrish C, Desir Y (2020). Toward universal HIV treatment in Haiti: time trends in ART retention after expanded ART eligibility in a national cohort from 2011 to 2017. J Acquir Immune Defic Syndr.

[40] Chi BH, Cantrell RA, Zulu I (2009). Adherence to first-line antiretroviral therapy affects non-virologic outcomes among patients on treatment for more than 12 months in Lusaka, Zambia. Int J Epidemiol.

[41] Fox MP, Rosen S (2010). Patient retention in antiretroviral therapy programs up to three years on treatment in sub-Saharan Africa, 2007-2009: systematic review. Trop Med Int Health.

[42] World Health Organization (2007). WHO Case Definitions of HIV for Surveillance and Revised Clinical Staging and Immunological Classification of HIV-Related Disease in Adults and Children.

[43] Barros AJD, Hirakata VN (2003). Alternatives for logistic regression in cross-sectional studies: an empirical comparison of models that directly estimate the prevalence ratio. BMC Med Res Methodol.

[44] Chen W, Qian L, Shi J, Franklin M (2018). Comparing performance between log-binomial and robust Poisson regression models for estimating risk ratios under model misspecification. BMC Med Res Methodol.

[45] Makeleni N, Cilliers L (2021). Critical success factors to improve data quality of electronic medical records in public healthcare institutions. SA Journal of Information Management.

[46] Kellerman S, Essajee S (2010). HIV testing for children in resource-limited settings: what are we waiting for?. PLoS Med.

[47] Rosen JG, Muraleetharan O, Walker A, Srivastava M (2023). Pediatric antiretroviral therapy coverage and AIDS deaths in the "Treat All" era. Pediatrics.

[48] Fetzer BC, Mupenda B, Lusiama J, Kitetele F, Golin C, Behets F (2011). Barriers to and facilitators of adherence to pediatric antiretroviral therapy in a sub-Saharan setting: insights from a qualitative study. AIDS Patient Care STDS.

[49] Wilkinson LS, Skordis-Worrall J, Ajose O, Ford N (2015). Self-transfer and mortality amongst adults lost to follow-up in ART programmes in low- and middle-income countries: systematic review and meta-analysis. Trop Med Int Health.

