# Supplementary figures and images for: Electronic Medical Record Data Missingness and Interruption in Antiretroviral Therapy Among Adults and Children Living With HIV in Haiti: Retrospective Longitudinal Study
Source: JMIR Pediatr Parent. 2024 Mar 6;7:e51574. doi: 10.2196/51574 (PMC10986334; doi:10.2196/51574)

Appendix 1. CONSORT flow diagram


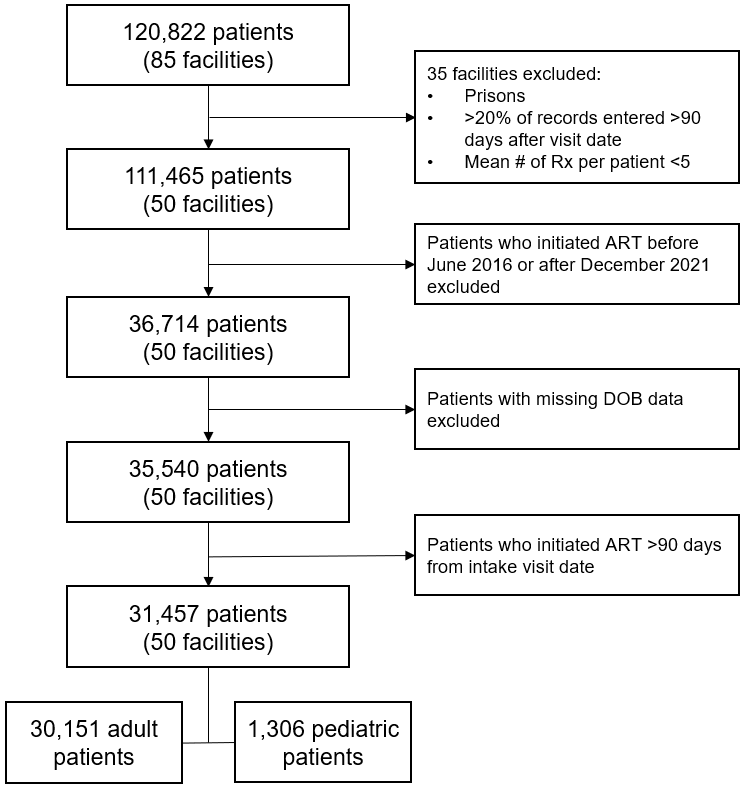

Supplement: Multimedia Appendix 1 [file pediatrics-v7-e51574-s001.docx]
