# Supplementary material for: Electronic Medical Record Data Missingness and Interruption in Antiretroviral Therapy Among Adults and Children Living With HIV in Haiti: Retrospective Longitudinal Study
Source: JMIR Pediatr Parent. 2024 Mar 6;7:e51574. doi: 10.2196/51574 (PMC10986334; doi:10.2196/51574)
Supplement: Multimedia Appendix 2 [file pediatrics-v7-e51574-s002.docx]

Supplemental Table 1. Multivariable regression of IIT status at 6 and 12 months post-ART initiation against composite missingness score (continuous), stratified by age group

|  | **Adult** | | | | | | **Pediatric** | | | | | |
| --- | --- | --- | --- | --- | --- | --- | --- | --- | --- | --- | --- | --- |
|  | **6-month IIT** | | | **12-month IIT** | | | **6-month IIT** | | | **12-month IIT** | | |
|  | **aRR** | **95% CI** | **p-val.** | **aRR** | **95% CI** | **p-val.** | **aRR** | **95% CI** | **p-val.** | **aRR** | **95% CI** | **p-val.** |
| Composite missingness score | 1.03 | (0.92, 1.15) | .579 | 1.02 | (0.94, 1.10) | .618 | 1.34 | (1.08, 1.66) | .008 | 1.24 | (1.05, 1.46) | .010 |
| Sex |  |  |  |  |  |  |  |  |  |  |  |  |
| Female | Ref | - | - | Ref | - | - | Ref | - | - | Ref | - | - |
| Male | 1.05 | (0.98, 1.11) | .158 | 1.02 | (0.97, 1.07) | .396 | 0.93 | (0.81, 1.08) | .335 | 0.99 | (0.88, 1.11) | .870 |
| Facility type |  |  |  |  |  |  |  |  |  |  |  |  |
| Health Center | Ref | - | - | Ref | - | - | Ref | - | - | Ref | - | - |
| Hospital | 0.84 | (0.60, 1.16) | .284 | 0.94 | (0.59, 1.49) | .795 | 0.86 | (0.64, 1.15) | .306 | 0.92 | (0.67, 1.26) | .609 |
| Dispensary | 0.81 | (0.56, 1.17) | .264 | 0.78 | (0.54, 1.11) | .166 | 1.13 | (0.79, 1.62) | .505 | 0.89 | (0.62, 1.28) | .533 |
| Facility ownership |  |  |  |  |  |  |  |  |  |  |  |  |
| Mixed | Ref | - | - | Ref | - | - | Ref | - | - | Ref | - | - |
| Private | 0.95 | (0.71, 1.28) | .757 | 1.04 | (0.74, 1.47) | .811 | 0.87 | (0.53, 1.44) | .589 | 0.87 | (0.60, 1.24) | .435 |
| Public | 0.90 | (0.65, 1.24) | .513 | 0.96 | (0.65, 1.42) | .832 | 0.91 | (0.69, 1.18) | .462 | 0.85 | (0.66, 1.10) | .224 |
| Duration of iSante use (years) | 1.02 | (0.97, 1.07) | .526 | 1.04 | (0.99, 1.08) | .120 | 0.98 | (0.93, 1.04) | .496 | 1.00 | (0.95, 1.04) | .874 |
| Monthly patient volume average (log) | 0.82 | (0.67, 1.00) | .046 | 0.74 | (0.58, 0.94) | .012 | 0.98 | (0.74, 1.28) | .863 | 0.92 | (0.75, 1.14) | .460 |

Supplemental Table 2. Multivariable regression of IIT status at 6 and 12 months post-ART initiation against composite missingness score (categorial), stratified by age group

|  | **Adult** | | | | | | **Pediatric** | | | | | |
| --- | --- | --- | --- | --- | --- | --- | --- | --- | --- | --- | --- | --- |
|  | **6-month IIT** | | | **12-month IIT** | | | **6-month IIT** | | | **12-month IIT** | | |
|  | **aRR** | **95% CI** | **p-val.** | **aRR** | **95% CI** | **p-val.** | **aRR** | **95% CI** | **p-val.** | **aRR** | **95% CI** | **p-val.** |
| Composite missingness score |  |  |  |  |  |  |  |  |  |  |  |  |
| 0 | Ref | - | - | Ref | - | - | Ref | - | - | Ref | - | - |
| 1 | 0.94 | (0.77, 1.15) | .570 | 0.96 | (0.85, 1.08) | .484 | 1.59 | (1.26, 2.01) | <.001 | 1.54 | (1.34, 1.78) | <.001 |
| 2 | 1.14 | (0.87, 1.51) | .343 | 1.11 | (0.91, 1.35) | .295 | 1.74 | (1.02, 2.97) | .041 | 1.34 | (0.82, 2.20) | .241 |
| 3 | 1.32 | (1.03, 1.70) | .030 | 1.21 | (0.95, 1.55) | .122 | 2.25 | (1.43, 3.56) | .001 | 1.75 | (1.08, 2.85) | .023 |
| Sex |  |  |  |  |  |  |  |  |  |  |  |  |
| Female | Ref | - | - | Ref | - | - | Ref | - | - | Ref | - | - |
| Male | 1.05 | (0.98, 1.12) | .196 | 1.02 | (0.97, 1.07) | .413 | 0.93 | (0.81, 1.06) | .291 | 0.99 | (0.86, 1.13) | .876 |
| Facility type |  |  |  |  |  |  |  |  |  |  |  |  |
| Health Center | Ref | - | - | Ref | - | - | Ref | - | - | Ref | - | - |
| Hospital | 0.84 | (0.57, 1.24) | .380 | 0.94 | (0.61, 1.46) | .795 | 0.85 | (0.61, 1.18) | .340 | 0.92 | (0.68, 1.25) | .605 |
| Dispensary | 0.82 | (0.56, 1.20) | .304 | 0.79 | (0.57, 1.09) | .144 | 1.16 | (0.86, 1.56) | .335 | 0.90 | (0.65, 1.25) | .541 |
| Facility ownership |  |  |  |  |  |  |  |  |  |  |  |  |
| Mixed | Ref | - | - | Ref | - | - | Ref | - | - | Ref | - | - |
| Private | 0.95 | (0.71, 1.26) | .719 | 1.04 | (0.74, 1.45) | .832 | 0.88 | (0.58, 1.35) | .566 | 0.87 | (0.60, 1.26) | .470 |
| Public | 0.90 | (0.68, 1.20) | .473 | 0.96 | (0.62, 1.48) | .852 | 0.92 | (0.72, 1.18) | .511 | 0.86 | (0.69, 1.08) | .195 |
| Duration of iSante use (years) | 1.01 | (0.97, 1.06) | .475 | 1.03 | (0.99, 1.08) | .098 | 0.98 | (0.92, 1.03) | .435 | 0.99 | (0.95, 1.04) | .796 |
| Monthly patient volume average (log) | 0.81 | (0.68, 0.97) | .022 | 0.74 | (0.60, 0.90) | .003 | 0.98 | (0.74, 1.30) | .882 | 0.91 | (0.74, 1.13) | .406 |

Supplemental Table 3. Multivariable regression of 6- and 12-month IIT against indicator missingness (binary, missing vs. non-missing), by age group

|  | **Adult** | | | | | | **Pediatric** | | | | | |
| --- | --- | --- | --- | --- | --- | --- | --- | --- | --- | --- | --- | --- |
|  | **6-month IIT** | | | **12-month IIT** | | | **6-month IIT** | | | **12-month IIT** | | |
|  | **aRR** | **95% CI** | **p-val.** | **aRR** | **95% CI** | **p-val.** | **aRR** | **95% CI** | **p-val.** | **aRR** | **95% CI** | **p-val.** |
| Weight missingness | 0.95 | (0.79, 1.14) | .556 | 1.01 | (0.83, 1.23) | .910 | 0.91 | (0.67, 1.25) | .564 | 0.87 | (0.63, 1.21) | .414 |
| WHO HIV stage missingness | 1.15 | (0.93, 1.41) | .193 | 1.03 | (0.92, 1.14) | .639 | 2.17 | (1.79, 2.64) | .001 | 1.79 | (1.54, 2.08) | <.001 |
| TB diagnosis missingness | 1.03 | (0.86, 1.24) | .749 | 1.03 | (0.87, 1.23) | .713 | 1.03 | (0.78, 1.38) | .821 | 1.01 | (0.65, 1.57) | .972 |
| Sex |  |  |  |  |  |  |  |  |  |  |  |  |
| Female | Ref | - | - | Ref | - | - | Ref | - | - | Ref | - | - |
| Male | 1.05 | (0.97, 1.12) | .217 | 1.02 | (0.97, 1.07) | .439 | 0.92 | (0.79, 1.06) | .238 | 0.98 | (0.86, 1.11) | .748 |
| Facility type |  |  |  |  |  |  |  |  |  |  |  |  |
| Health Center | Ref | - | - | Ref | - | - | Ref | - | - | Ref | - | - |
| Hospital | 0.83 | (0.58, 1.21) | .335 | 0.94 | (0.60, 1.47) | .788 | 0.83 | (0.65, 1.07) | .148 | 0.89 | (0.70, 1.12) | .318 |
| Dispensary | 0.81 | (0.56, 1.18) | .275 | 0.78 | (0.55, 1.11) | .162 | 1.14 | (0.88, 1.48) | .335 | 0.92 | (0.69, 1.23) | .574 |
| Facility ownership |  |  |  |  |  |  |  |  |  |  |  |  |
| Mixed | Ref | - | - | Ref | - | - | Ref | - | - | Ref | - | - |
| Private | 0.95 | (0.72, 1.26) | .739 | 1.04 | (0.75, 1.45) | .804 | 0.97 | (0.73, 1.30) | .850 | 0.90 | (0.68, 1.19) | .456 |
| Public | 0.90 | (0.65, 1.25) | .541 | 0.96 | (0.64, 1.42) | .834 | 1.00 | (0.78, 1.28) | .979 | 0.89 | (0.72, 1.11) | .303 |
| Duration of iSante use (years) | 1.01 | (0.97, 1.06) | .596 | 1.04 | (0.99, 1.08) | .135 | 0.98 | (0.94, 1.02) | .302 | 0.99 | (0.95, 1.04) | .760 |
| Monthly avg. patient volume (log) | 0.82 | (0.68, 1.00) | .052 | 0.74 | (0.60, 0.91) | .004 | 0.94 | (0.77, 1.15) | .543 | 0.91 | (0.75, 1.10) | .336 |
